# Research among undergraduate biomedical students in Cameroon: contextual barriers, room for improvement

**DOI:** 10.11604/pamj.2019.33.149.18807

**Published:** 2019-06-27

**Authors:** Etienne Ngeh Ngeh

**Affiliations:** 1 Research Organization for Health Education and Rehabilitation-Cameroon (ROHER-CAM), Bamenda, Cameroon; 2 Physiotherapy Department, St. Louis University Institute of Health and Biomedical Sciences-Bamenda, Bamenda, Cameroon; 3 Physiotherapy Service, Regional Hospital Bamenda, Bamenda, Cameroon

**Keywords:** Biomedical students, health research, undergraduate, Cameroon

## To the editors of the Pan African Medical Journal

There is overwhelming evidence that the highest burden of disease worldwide is concentrated in low and middle-income countries (LMICs) and responsible for the high mortality in these countries [1]. This high mortality rates have strongly been associated with poor health care systems and sub-optimal quality of health care delivery. The poor quality of health care delivery is powered by a lack of research evidence [2]. Evidence can only be gotten by conducting high-quality research and generating local data based on peculiar health problems that can be used to inform guidance [3, 4]. Encouraging research among students early during training has been an effective strategy in stimulating interest in future research [5, 6]. This correspondence highlights some contextual barriers to clinical research practice among undergraduate biomedical students in Cameroon and suggests ways to improve the situation.

### Barriers

The existing contextual barriers to undergraduate research are complex and interrelated and can include:

**Poor research infrastructure:** poor research production in Cameroon can grossly be associated to the weak and unavailable functional research infrastructures at the institutional level. Most public and private institutions lack the commitment to investment in research structures, making research practice unrealistic with poor student referral and uptake into health research.

**Poor study facilities:** undergraduate training institutions in Cameroon are substandard with inadequate training facilities. Observed in these institutions are outdated and under-resourced library stocks, lack of computers, poor internet and e-libraries limiting access to research literature. Good research questions, protocols, appraisal and review of current literature are unrealizable without easy access to the literature.

**Lack of proper mentorship:** the observed attitude, knowledge, and practices of faculty lead, and those assuming research supervisory roles in our undergraduate training institutions in Cameroon leaves little to be desired by students. The missing track record of many providing guidance leaves students with a univocal view of research as a graduation requirement. This attitude has left students with the inability to appraise and engage in health research. The situation is further compounded by incompetent assessors, whose main motivation is pecuniary rather than the core values of the supposed exercise.

**Late introduction of students to the research process:** all undergraduate training programs for biomedical students tend to have a research course. The course is often introduced late during training and directed towards the final year project or thesis. Coupled with tight training programs, students work within a short time under pressure on research projects. The associated pressure and lack of clarity of the research process have led many to hate research.

**Lack of funding and promotion:** high-quality research is unthinkable without appropriate funding. Lack of institutional and national frameworks to promote research among young researchers and students also halts the promotion of research culture in Cameroon. Many faculties and supervisors lack funded projects that may enroll students into practical research during training.

**Lack of journals:** the rare availability of institutional and national journals coupled with the financial obligations for publications by journals constitute research barriers. Furthermore, some international journals rarely accept manuscripts originating from lesser well-known authors [2].

### Suggestions

To improve health research in undergraduates, it is mandatory to reconsider making positive changes to the presenting of contextual barriers. Strengthening existing research infrastructures and creating new ones from the national framework to operational research units at the clinical site is non-elective. This may be a good investment to target health problems effectively in the near future. Such establishments may engage interested students early into clinical research. Accreditation/supervision of training institutions for biomedical students should be comprehensive and rigorous. All approved institutions should meet the minimum training standards with all the necessary study facilities for high quality training and research. Only adept enthusiastic teachers/lecturers should be assigned for research supervision. Hence, training institutions may make it as a target for all lecturers assuming a supervisory role to publish on a regular basis as this may naturally benefit the students as they advance their career. There is a need to revisit our crowded training programs with early introduction of the research process to allow graduates to embrace the tenets of research early during training. Early understanding of the research process may translate to increase research engagement during training and in future. Early enrollment of students into research and journal clubs in biomedical institutions may further consolidate this research attitude. Such units may look for partnership with active collaborators to promote research [2]. Promotion of student participation in scientific conferences and journal publications may motivate and engage students into research. This may include providing travel grants for students with good abstracts and research articles for congresses and conferences. This may convey the feeling of accomplishment and stimulate future engagement in research and publication [5]. Financially stable stakeholders and organizations should be educated to play the role of donor agencies for achievable research for biomedical students [7]. Creation of small grants and engagement of students by supervisors are viable ways to improve research funding for students.

## Conclusion

Barriers to undergraduate biomedical students' research in Cameroon can be much improved if training institutions invest into research. Practical efforts to changing student attitude towards research from a graduation requirement to a problem solving skill during training are necessary.

## Competing interests

The author declares no competing interests.
